# Photodynamic therapy in brain cancer: mechanisms, clinical and preclinical studies and therapeutic challenges

**DOI:** 10.3389/fchem.2023.1250621

**Published:** 2023-11-24

**Authors:** Wojciech Domka, Dorota Bartusik-Aebisher, Izabela Rudy, Klaudia Dynarowicz, Karolina Pięta, David Aebisher

**Affiliations:** ^1^ Department of Otolaryngology, Medical College of the University of Rzeszów, Rzeszów, Poland; ^2^ Department of Biochemistry and General Chemistry, Medical College of the University of Rzeszów, Rzeszów, Poland; ^3^ Students English Division Science Club, Medical College of the University of Rzeszów, Rzeszów, Poland; ^4^ Center for Innovative Research in Medical and Natural Sciences, Medical College of the University of Rzeszów, Rzeszów, Poland; ^5^ Department of Photomedicine and Physical Chemistry, Medical College of the University of Rzeszów, Rzeszów, Poland

**Keywords:** photodynamic therapy, brain cancer, photosensitizers, clinical studies, challenges

## Abstract

Cancer is a main cause of death and preferred methods of therapy depend on the type of tumor and its location. Gliomas are the most common primary intracranial tumor, accounting for 81% of malignant brain tumors. Although relatively rare, they cause significant mortality. Traditional methods include surgery, radiotherapy and chemotherapy; they also have significant associated side effects that cause difficulties related to tumor excision and recurrence. Photodynamic therapy has potentially fewer side effects, less toxicity, and is a more selective treatment, and is thus attracting increasing interest as an advanced therapeutic strategy. Photodynamic treatment of malignant glioma is considered to be a promising additional therapeutic option that is currently being extensively investigated *in vitro* and *in vivo*. This review describes the application of photodynamic therapy for treatment of brain cancer. The mechanism of photodynamic action is also described in this work as it applies to treatment of brain cancers such as glioblastoma multiforme. The pros and cons of photodynamic therapy for brain cancer are also discussed.

## 1 Introduction

### 1.1 Brain cancer

Malignant gliomas are the second most common cause of cancer deaths among people under 35 years of age (Almammadov et al., 2022). Brain tumors can be divided into primary tumors, i.e., those that arise directly from the brain tissue, and metastatic tumors, which develop in other organs and spread to the brain (Kim and Lee, 2022). There are over 120 types of brain tumors, and approximately 45% of primary brain tumors are gliomas, the most common and aggressive of which is glioblastoma multiforme (GBM) (Kharroubi Lakouas et al., 2017). The classification system developed by the World Health Organization (WHO) is based on the histological features of brain tissues observed under a microscope and divides brain tumors into malignant and benign tumors (Kim and Lee, 2022). The treatment of brain tumors still poses many challenges, and one of the main limitations is the problem of drug delivery to the brain due to the presence of the selective blood-brain barrier formed by endothelial cells of the cerebral vessels which regulates the transport of nutrients and ions protecting the brain from neurotoxic molecules. For this reason, most drugs cannot cross the blood-brain barrier by physiological routes (Kim and Lee, 2022). One of the most commonly used methods of glioblastoma therapy is chemotherapy, which is characterized by high cytotoxicity, affecting both tumor cells and healthy cells of the body. Chemotherapy does not have the ability to distinguish cancer cells from healthy cells (Ladomersky et al., 2019; Meng et al., 2022). A common phenomenon in the treatment of brain gliomas is their multidrug resistance (Meng et al., 2022). The mainstay of treatment of high-grade brain tumors is still surgical resection in combination with radiotherapy and chemotherapy. The invasive growth pattern, especially in certain areas of the brain, often makes it difficult to completely remove the tumor and it is important to realize that not all of the tumor tissue will be resected. This forces us to search for new methods of brain tumor therapy, which will be characterized by both safety for the patient and high efficiency (Quirk et al., 2015). The healing potential of light was noticed in antiquity, when the treatment of various ailments, ranging from mood disorders to musculoskeletal disorders and skin diseases, was treated by exposing the patient to sunlight.

### 1.2 Photodynamic therapy (PDT)- mechanism

**FIGURE 1:**
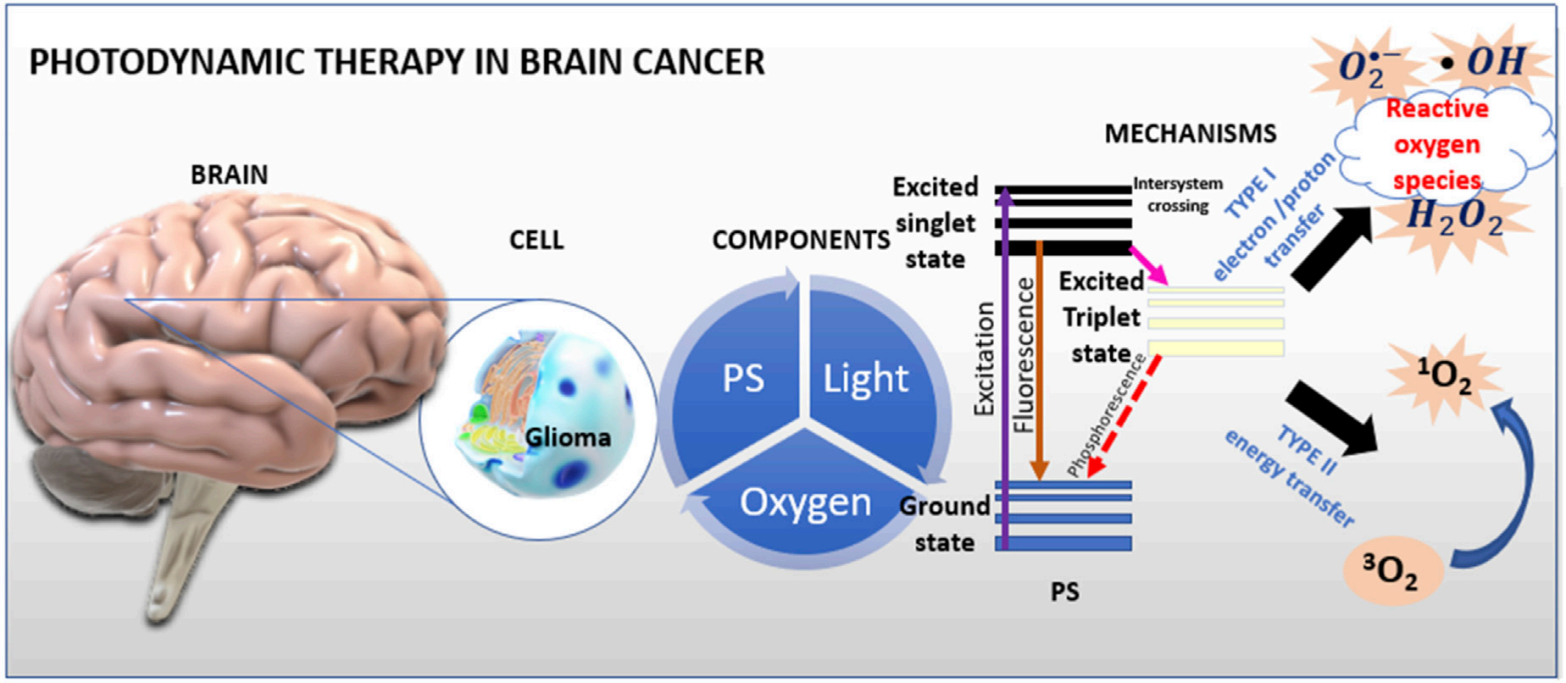
Mechanism of PDT. Photosensitizers accumulate in diseased cells and then are irradiated with laser light or visible light During light absorption, the accumulated PS molecules in the cells pass from the singlet ground state to the excited singlet state. Much of the energy is lost through direct photon emission in the excited state through fluorescence. A small part of the energy is used in the process of intersystem crossing to the excited triplet state. The active form of the PS in the triplet state, reacts with oxygen causing the formation of active oxygen species, i.e., singlet oxygen. These molecules induce apoptosis - programmed cell death. Figure 1 shows a schematic of photodynamic action. Interpretations of the entire procedure can be reduced (simplified) to the statement that PDT is the conversion of light energy into toxic, reactive oxygen species.

Photodynamic therapy (PDT) was first discovered over a century ago by medical student Oscar Raab, working with Professor Hermann von Tappeiner, who was the first to use the term “photodynamic therapy” (Niculescu and Grumezscu, 2021). Photodynamic therapy was originally developed as a treatment target for dermatological diseases. Skin lesions are easily exposed to light and are easily monitored by visual assessment. Thanks to the successes in the treatment of skin diseases, the so-called interstitial photodynamic therapy (iPDT), a promising treatment for deep tumors using optical fibers inserted *in situ* where targeting strategies aim to improve the delivery of photosensitizers (PS) to tumor tissues to simultaneously increases the selectivity and effectiveness of PDT are being sought. Photodynamic therapy is currently a method of treating various disease cases (Gunaydin et al., 2021). The main principle of operation is based on the localization of the PS molecule within cancer cells (Oniszczuk et al., 2016). The first stage is the excitation of the inflammatory/neoplastic area with light of a specific wavelength in the presence of oxygen and the PS (Baskaran et al., 2018; Kwiatkowski et al., 2018). Reactive products such as singlet oxygen and hydroxyl radical are generated at the light-exposed site (dos Santos et al., 2019). These free radicals interact with molecules of lipids, peptides, proteins and nucleic acids (Mansoori et al., 2019). They are able to directly kill cancer cells by apoptosis and necrosis, damage tumor vascular structures leading to hypoxia, and induce immune responses (Gunaydin et al., 2021). The group of reactive PDT products includes oxygen free radicals that are formed as a result of electron transfer (e.g., superoxide anion O_2_^•−^, hydroxyl radical HO•, hydroperoxyl radical HOO•), and as a result of energy transfer, singlet oxygen is obtained. Apart from light and oxygen, PS are an important component of photodynamic therapy. Figure 1 shows mechanism of PDT in brain cancer.

Photodynamic therapy involves administering PS that accumulates in the patient’s tissues. A PS is a non-toxic substance that has no negative effects on health in its inactive state. However, the PS exposed to irradiation becomes a source of free oxygen radicals that destroy surrounding cells. This means that the drug will act locally only in the area where it was activated by light, sparing healthy tissue. The described therapy model is already used in the case of numerous diseases, e.g., dermatological diseases or cancers and pre-cancerous lesions of the esophagus, and recently also in the treatment of brain tumors. Singlet oxygen is extremely reactive and has the ability to damage cellular proteins as well as DNA itself (Kostron, 2010; Agnez-Lima et al., 2012; Kwiatkowski et al., 2018; Chizenga et al., 2019; Fujii et al., 2023; Murotomi et al., 2023).

The most common features of a good PS are high selectivity towards the tissue we want to work on and high cytotoxicity in the presence of light (Dolmans, et al., 2003). Research is continuing on the search for new, more organ-specific PS. However, before they are introduced into practice, they must undergo a series of clinical trials (Dougherty and Marcus, 1992; Farokhzad and Langer, 2009). The type and concentration of the PS used, as well as the wavelength of the light source used, affect the extent and type of damage caused by PDT (Agostinis et al., 2011). In terms of superficial lesions on the mucous membranes and skin, the effectiveness of PDT has been clinically demonstrated and therefore it has already been introduced in the clinic (Lamberti et al., 2014; Bacellar et al., 2015; Baptista et al., 2017). Unfortunately, lesions located deeper in the tissues are still a problem because access to them is difficult, and the light also has a limited penetration depth (Zhao et al., 2018; Sun et al., 2019). The search for new, more tissue-specific PS is constantly ongoing. Research is also conducted on the modification of already available photosensitizing substances in order to minimize their effect on specific organs and eliminate the potentially harmful effect of PDT on healthy tissues (Thomas et al., 2017).

Photodynamic therapy is considered a less invasive therapy targeting all cells that contain the PS itself, while preserving adjacent normal tissues (Quirk et al., 2015; Akimoto, 2016; Leroy et al., 2021; Bartusik-Aebisher et al., 2022). Due to the nature and type of products generated during PDT, it has cytotoxic properties (Santos et al., 2020). Due to the extraordinary therapeutic properties of the method, this review discusses the mechanism of phototherapy, the application of the presented method (mainly in diseases of the nervous system and brain), and determining its advantages and disadvantages, both in preclinical and clinical trials (Agostinis, et al., 2011).

## 2 Methodology

**FIGURE 2:**
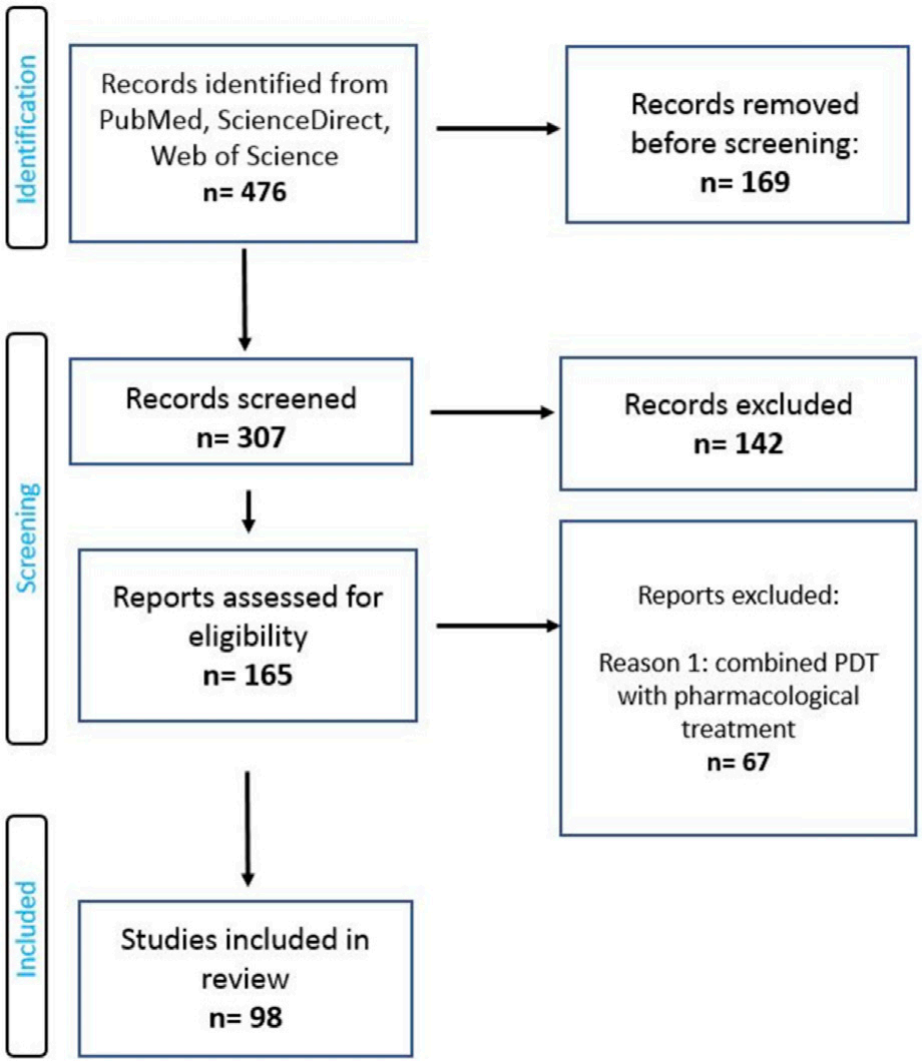
PRISMA flow diagram of included studies.

In this paper, we reviewed the current preclinical and clinical studies showing the use of PDT in the treatment of brain tumors. Articles for individual sections have been collected from the following scientific databases: PubMed, ScienceDirect, Web of Science and Google Scholar. The main part presents an overview of *in vitro* and *in vivo* studies of PDT in brain tumors. Research papers, review papers and short essays from inception to 2023 were qualified for the review. The qualification scheme of individual articles is presented in Figure 2.

Additionally, the PICO criteria used for the selection of literature are presented in Table 1. The PICO criteria were: population, intervention, comparison and outcome of the research.

**TABLE 1:** PICO framework.

PICO Question	
Problem	Research object: patients with brain diseases
Intervention	Application of a theory or method; treatment with PDT, an overview of the most commonly used photosensitizers
Comparison	Alternative theories or methods (or, in their absence, the null hypothesis); review not only clinical trials but also pre-clinical *in vitro* studies
Outcome	Knowledge generation: increase in the effectiveness of PDT

## 3 Application of photodynamic therapy in the treatment of brain tumors

### 3.1 Applied photosensitizers (PS)

The term “photosensitizer” means a substance that sensitizes the test site to light. There are many different PS known in medicine, which differ primarily in the extent of laser light absorption. In medicine, PS that cause more apoptosis and less inflammation are extremely desirable (Agostinis, et al., 2011). The key function of PDT is the ability to influence the immune system. The inflammatory response and necrotic cell death is one of the effects of PDT that characterizes the effectiveness of the therapy.

There are many different PS currently being tested and used in clinical trials (including in the treatment of brain tumors) (Mahmoudi et al., 2019). The PS is characterized by such properties as: toxicity, time and effectiveness of concentration in the tissue, the range of light absorption and the depth of tissue penetration (Correia et al., 2021). At the moment, there are no PS that meet all aspects, mainly in the treatment of brain diseases (Quirk et al., 2015).

Photosensitizers have been divided into three generations; the former consists of naturally occurring porphyrins, including hematoporphyrin. This group of molecules is characterized by strong absorption at a wavelength of about 400 nm. The second generation of PS includes compounds such as sodium thalaporfin and temoporfin, and 5-aminolevulinic acid (5-ALA), activated by wavelengths greater than 600 nm (Cramer and Chen, 2020). Third-generation PS are components of second-generation PS that include a nanoelement (Niculescu and Grumezescu, 2021). The first clinically approved PS was porfimer sodium, sold under the trade name Photofrin^®^. It is used in the treatment of non-small cell lung cancer, bladder cancer, esophageal cancer and brain cancer. Despite their wide application in medicine, first-generation PS are characterized by a number of unfavorable features; they have a low chemical purity and can be activated at wavelengths below 640 nm. Another disadvantage is their long half-life, which makes the skin hypersensitive to light. Patients treated with these PS are required to stay in a dark room for up to 6 weeks (Niculescu and Grumezescu, 2021). The absorption spectrum of PS belonging to the second generation is in the range of 650–800 nm, which allows for better absorption of light in deep tissue. Thanks to faster elimination from the body, they are characterized by fewer side effects and a shorter period of photosensitivity. The disadvantage of these compounds is their hydrophobicity, which is the reason for the aggregation of second-generation PS under physiological conditions, which in turn is the reason for the reduction of ROS production efficiency. The hydrophobic nature of these compounds also limits their intravenous administration, which forces the search for new methods of drug delivery (Kharroubi Lakouas et al., 2017; Park et al., 2018)]. Third generation photosensitizers are more stable and hydrophilic. They have favorable pharmacodynamic and pharmacokinetic parameters and are characterized by better biodistribution *in vivo* although as yet, no third generation PS is approved for clinical use. They have fewer side effects and less toxicity (Guo, et al., 2015).

The characteristics of the most popular photosensitizers used in PDT of brain tumors are presented below.

#### 3.1.1 Porfimer sodium, photofrin, HPD

**FIGURE 3:**
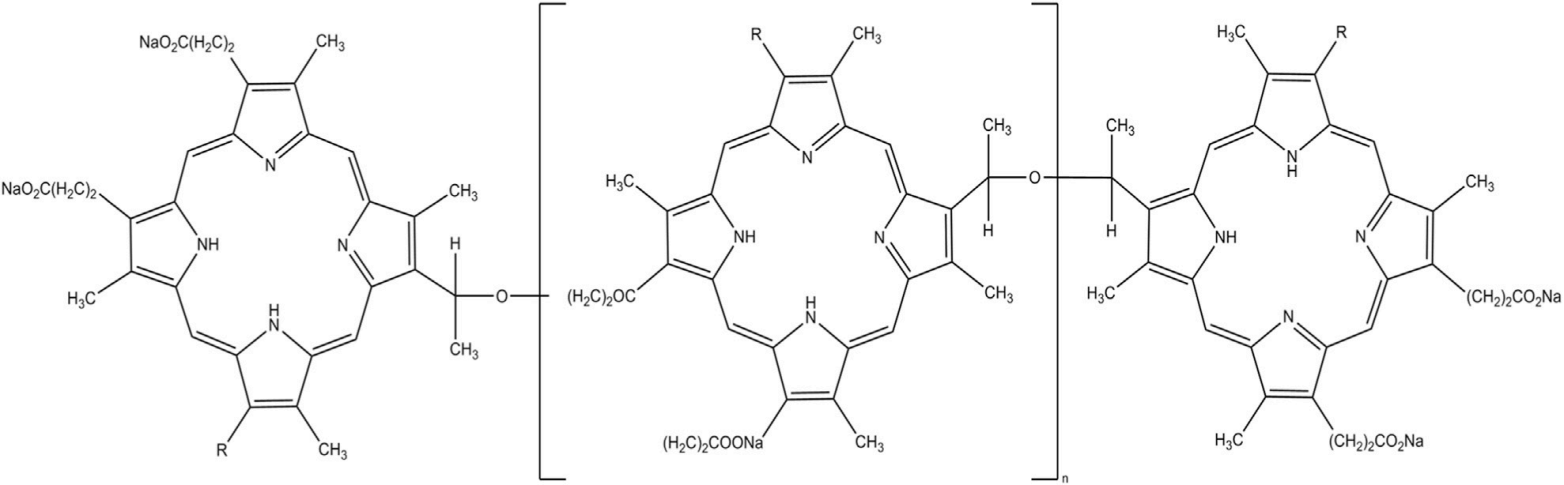
Structure of porfimer sodium.

The first PS used clinically for the treatment of cancer was a hematoporphyrin derivative, now known as Photofrin^®^ (Agostinis et al., 2011). This hematoporphyrin derivative has been used for PDT in oncology for the treatment of thousands of patients around the world for 40 years. Various forms are currently available on the market (Allison and Sibata, 2010). Sodium porfimer has an absorbance in the range of 630 nm [14]. Jiang studied the effect of PDT in combination with Photofrin^®^ on human glioblastoma cell lines. The light source was a laser with a wavelength of 632 nm and the photosensitizer Photofrin^®^ was applied in different doses. The aim of the study was to determine the degree of migration of glioblastoma cells post-PDT. The results of the experiment showed that PDT with Photofrin^®^ inhibited the spread of cells lines U87 and U25ln. The authors concluded that PDT in combination with Photofrin® neutralizes and blocks the development and dynamics of glioblastoma cells (Jiang et al., 2002). In another study by Jiang et al., PDT was used in combination with liposome encapsulated Photofrin^®^ in a human glioblastoma cell line injected into nude rats. The results of the experiment confirmed that PDT with the application of Photofrin^®^ in a liposome led to greater tumor destruction. The conclusions of the study were as follows: PDT in combination with Photofrin^®^ can aid in the treatment of brain tumors in humans (Jiang F et al., 1998)]. In turn, deCarvalho et al., applied PDT with Photofrin^®^ to glioblastoma cells. In this experiment, the applied therapy changed the homeostasis of the tumor, becoming a suitable model to control the effect of PDT in the treatment of glioblastoma (deCarvalho et al., 2007). Photofrin^®^ was also used in research by Lee et al. In their experiments, the authors studied the mechanisms of the cellular response in glioblastoma cells (Lee et al., 2010). In preclinical studies, a group of dogs was also noted as a study group in PDT. Whelan et al., performed PDT on a group of dogs with and without brain tumor disease (control group). Photofrin^®^ was administered intravenously and exposed to light. The study showed that low doses of the photosensitizer initiated an apoptotic tumor response without significant persistent toxicity (Whelan et al., 1993). Figure 3 shows the structure of porfimer sodium.

#### 3.1.2 5-aminolevulinic acid, 5-ALA

5-aminolevulinic acid (5-ALA) is a biosynthetic precursor of protoporphyrin IX and is a second generation PS. ALA in structure and physiology is analogous to amino acids. It is rapidly absorbed and then converted to heme in normal cells through a metabolic pathway. It is assumed that in various cancer cells, exogenous application of larger amounts of ALA increases the cellular level of PpIX, which results in greater accumulation of PpIX in cancer cells than in normal cells. One of the better examples of an article describing the process of transformation of 5-ALA into PpIX is the work by Kennedy and Pottier (Kennedy and Pottier, 1992). According to the authors and their review, after the application of 5-ALA there is increased activity of the bile ducts. Transport of the compound into the intestines induces dissemination to most organs and tissues. In turn, administration of 5-ALA to animals (mice) induces PpIX fluorescence in the parotid glands. Under constant and normal conditions, the process of heme synthesis is dependent on the process of its removal from the body. The liver, as the main site of heme synthesis, works on the principle of feedback. In this process, the presence of free heme inhibits the synthesis of 5-ALA. The dynamic and efficient course of heme removal and synthesis causes a reduction in the amount of free heme in the liver, so that the synthesis of 5-ALA will not be inhibited, on the contrary, the amount and concentration will increase. The increase in 5-ALA concentration contributes to the increase in heme synthesis. Therefore, under physiologically constant and normal conditions, the demand for heme controls the rate of heme synthesis and therefore the rate of PpIX synthesis. Currently, there are many steps that induce the synthesis of 5-ALA and heme. As previously mentioned, the main site of synthesis is the liver, but the feedback mechanism is also present in other tissues. Uroporphyrins I and III and coproporphyrin I and III are strong tissue sensitizing substances. They come from uroporphyrinogen and coproporphyrin from coproporphyrinogen, i.e., from the heme biosynthesis pathway, which leads to the accumulation of porphyrins. In the metabolic pathway of 5-ALA synthesis, each of the intermediates has a sufficient power reserve, without accumulation. This does not happen when the concentration is not controlled and the amount of exogenous 5-ALA is high. The rate of synthesis of the first intermediate in the metabolic pathway (porphobilinogen) depends primarily on the maximum efficiency of the enzyme system responsible for this particular step. Further stages are controlled and conditioned by the other processes involved. The authors report that if porphobilinogen is synthesized at a rate that exceeds the maximum capacity of the next step in the pathway (in this case, uroporphyrinogen synthesis), then porphobilinogen will accumulate. On the other hand, however, if the mechanism responsible for the synthesis of uroporphyrinogen still has some reserves even though porphobilinogen is produced at the maximum possible rate, then the rate of uroporphyrinogen synthesis will be limited by the rate of porphobilinogen synthesis. The rule of this principle is applicable to each subsequent step of the biosynthetic metabolic pathway. If the first step of decarboxylation of uroporphyrinogen is slower than the synthesis of uroporphyrinogen, then the accumulation of uroporphyrinogen and, subsequently, uroporphyrin will occur. If the conversion of PpIX to heme is slower than the rate of PpIX synthesis, then PpIX will accumulate. It is worth noting that although the maximum capacity to synthesize PpIX from protoporphyrinogen may greatly exceed the maximum capacity of the next iron-dependent step in which PpIX is converted to heme, PpIX will not accumulate if the even slower process is located anywhere upstream of PpIX in the heme biosynthetic pathway. The presence or absence of such a rate-limiting step may explain why only certain cell types accumulate PpIX when exposed to high concentrations of exogenous ALA.

ALA is analogous to amino acids in terms of structure and physiology. It is rapidly absorbed and then metabolically converted to heme in normal cells. It is assumed that in various cancer cells, exogenous administration of larger amounts of ALA increases the cellular level of PpIX, which results in greater accumulation of PpIX in cancer cells than in normal cells. It is worth noting, however, that the mechanism of accumulation and synthesis of PpIX from ALA is still not fully understood.

**FIGURE 4:**
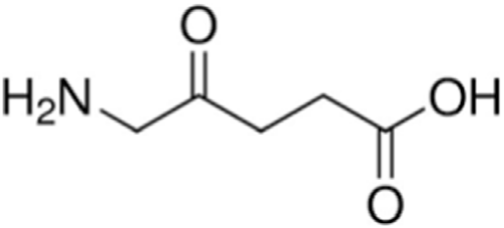
Structure of 5-aminolevulinic acid.

The maximum absorbance of this PS occurs at 630 nm (Agostinis et al., 2011). Currently, 5-ALA is one of the most commonly used photosensitizers in PDT. According to Mahmoudi et al., the application of 5-ALA in PDT improves tumor visibility through a specific metabolic process (Mahmoudi et al., 2019). Also, Lietke et al. applied 5-ALA to interstitial PDT (iPDT). The generated immune response and the production of reactive oxygen species resulted in a local cure of the disease. Interstitial photodynamic therapy is an innovative therapeutic approach consisting of direct introduction of light to the source of the tumor. 5-ALA is very often used in this method. At the moment, the use of 5-ALA is safe and effective in the treatment of brain tumors (Lietke et al., 2021). 5-ALA causes tumor cells to have a higher accumulation of protoporphyrin IX. According to Stepp and Stummer, the combination of 5-ALA with PDT is an innovative and non-toxic method of treatment (Stepp and Stummer, 2018). Due to its safety and negligible number of side effects, 5-ALA is also used in pediatric patients. *In vitro* studies conducted by Schwake et al. demonstrated that it is safe enough to be used as an agent to reduce the number of glioblastoma cells in children (Schwake et al., 2018). Omura et al., evaluated the efficacy of PDT in combination with 5-ALA on a human glioblastoma cell line. After the therapy, the effectiveness of the therapy was noticeable in all cell lines. The cell lines showed a lower tendency to tumorigenesis (Omura et al., 2023). Figure 4 shows the structure of 5-aminolevulinic acid.

#### 3.1.3 Temoporfin (Foscan), mTHPC

Chlorins belong to the group of tetrapyrrole macrocycles. These photosensitizers are considered one of the most effective photosensitizers used in PDT. They absorb light in the range from 650 to 750 nm. They also have high quantum efficiency and generate large amounts of ROS, including singlet oxygen (Mata et al., 2023).

**FIGURE 5:**
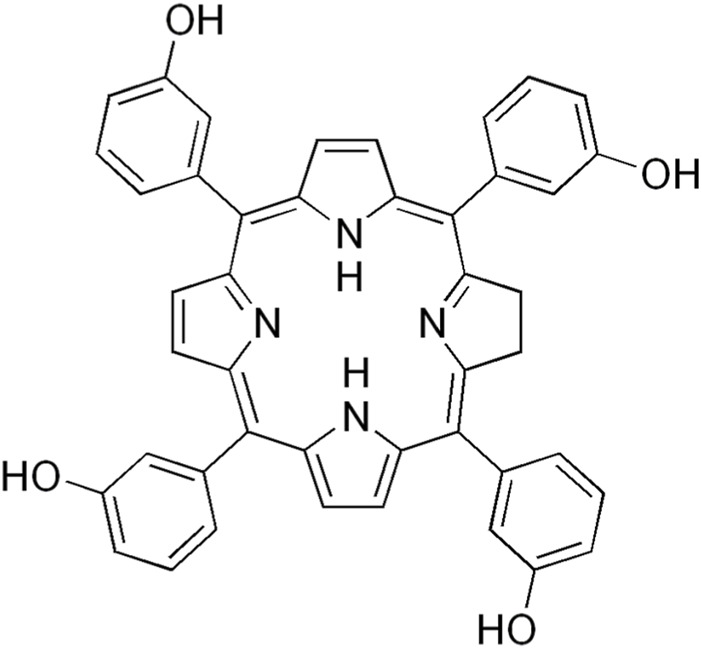
Structure of temoporfin.

Temoporfin (Foscan^®^) is commercially available and is one of the strongest PSs and is most efficient at an absorption of 652 nm (Allison and Sibata, 2010). Temoporfin is one of the stronger photosensitizers used in PDT. Yakavets et al. reviewed the efficacy of PDT with temoporfin. The review confirmed that nanoplatforms used in conjunction temoporfin contribute to an increase in the effectiveness and penetration of treatment during PDT. This fact is an extremely promising treatment opportunity compared to conventional treatments (Yakavets et al., 2019). One of the challenges of the continued use of temoporfin in PDT in the treatment of brain disorders is relatively low water solubility. These limitations suggest that in some studies the production of reactive oxygen species is not sufficient (Marconi et al., 2023). There is as yet scant research describing the use of temoporfins in the treatment of brain diseases. Laboratory tests are still needed to analyze the effect of this photosensitizer on brain cancer cells. An example of such a study is the work of researchers Bœuf-Muraille et al. In this study, a temoporfin-containing complex applied to a murine glioblastoma cell line was analyzed. The encapsulated photosensitizer had a higher toxicity compared to the uncomplexed photosensitizer. In their conclusions, the authors showed that the main pathway of cancer cell destruction was apoptosis (Bœuf-Muraille et al., 2019). Another problem or challenge in the use of temoporfins in clinical trials is the problem of patient photosensitivity after therapy. One solution is to develop a method of delivering the photosensitizer inside the tumor. Mannino et al. used rats previously implanted with a tumor in their study. The authors confirmed that the application of complexed photosensitizer is more effective, because the time to reach the optimal concentration is shorter compared to the systemic application (Mannino et al., 2008). In a study by Vanacloch, et al., temoporfin was used as a second-generation photosensitizer after Photofrin^®^. During therapy with temoporfin, a laser with a wavelength of 652 nm was used. The photosensitizer itself was administered intravenously. Compared to the previous photosensitizer, PDT with temoporfin penetrated deeper into the brain tissue and the washout time from the body was shorter (Vanaclocha et al., 2015). Figure 5 shows the structure of Temoporfin.

#### 3.1.4 Other chlorins

Chlorin e6 is also one of the strongest photosensitizers with low toxicity and quick dissolution and clearance from the body. According to the literature, this photosensitizer also has strong fluorescent properties. Due to the presented advantages, chlorin e6 is one of the most effective photosensitizers used in PDT (Shrestha et al., 2022).

PDT combined with chlorin e6 has antibacterial, anti-inflammatory and antimicrobial properties (Hur et al., 2022). Due to the fact that it belongs to the chlorine group of photosensitizers, it has no dark toxicity and is quickly removed from the body (Thapa Magar et al., 2023).

One of the disadvantages of chlorin e6 is its hydrophobicity, which correlates with poor biodistribution, consequently leading to the rapid removal of the photosensitizer from the body’s circulatory system (Hak et al., 2023).

According to Tereshkin et al., chlorin e6 also has other limitations, e.g., chlorin e6 is characterized by poor solubility in water, which leads to ineffective pharmacokinetics. Additionally, its effectiveness is lower compared to photosensitizers of the same generation. Moreover, this photosensitizer has a limited penetration depth (Tereshkina et al., 2022).

Chlorin e6 is the basic ingredient of talaporfin. Like other photosensitizers, chlorin e6 is used in the treatment of brain tumors. Teng et al., constructed cyanine-chlorine nanoclusters for the treatment of glioblastoma multiforme (Teng et al., 2020). They used a cell line that was administered an amount of photosensitizer and exposed to laser light. The study also included orthotopic models. The study showed that the concept of treating malignant gliomas, including fluorescence-guided resection with PDT adjuvant, is possible and feasible using the theragnostic nanocluster component.

#### 3.1.5 Talaporfin (LS11, MACE, NPe6)

**FIGURE 6:**
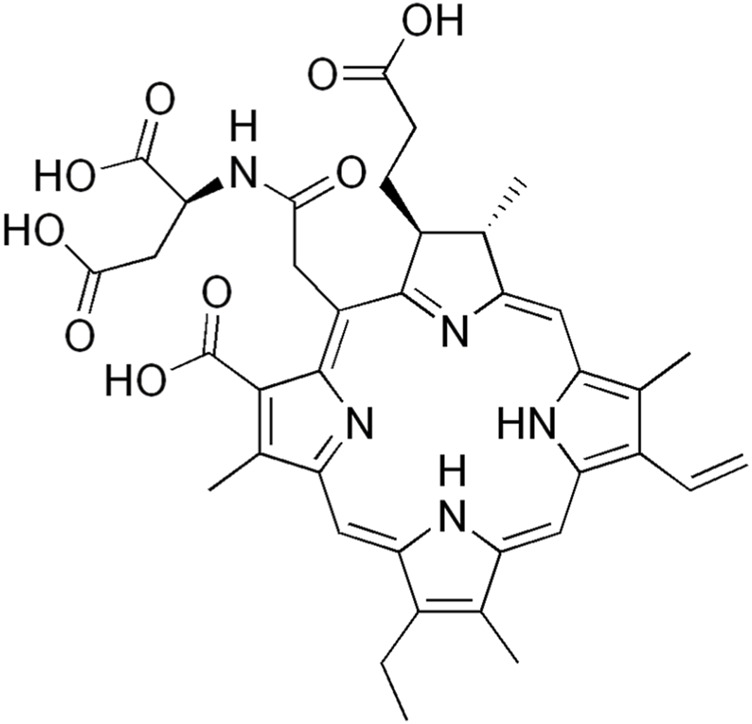
Structure of talaporfin.

Talaporfin is chlorin derivative and has many names including MACE, NPe6, LS11. The light absorption range of talaporfin is in the range of 664 nm. It is worth noting that the accumulation time of PS in the tissue is about 4 h (Allison and Sibata, 2010). Muragaki, et al., in their study used talaporfin sodium for PDT in patients with malignant parenchymal changes in brain tumors. The research group consisted of 22 patients with a diagnosed and confirmed disease. According to the authors, the intraoperative form of PDT may prove to be an effective alternative therapeutic method. In addition, at the moment it turns out to be safe. The use of this type of therapy in the near future may significantly affect the therapy of patients with brain tumors (Muragaki et al., 2013). Another example is a study was that conducted by Namatame et al. Their experiment used a rat model in which glioblastoma cells were injected into the frontal lobe. A diode laser with a wavelength of 664 nm was used as the light source. The results of the experiment were as follows: the authors observed necrosis and a decrease in the migration of tumor cells. A few hours after treatment, increased activity and expression of cells responsible for cell death were noted. The applied therapy was the cause of the progressive apoptosis process in the glioblastoma cells (Namatame et al., 2008). Similar conclusions were presented in work by Akimoto (Akimoto et al., 2012), who stated that PDT in combination with talaporfin is a milestone and at the same time a breakthrough in the therapy of glioblastoma. Due to the selectivity of the therapy, it is a promising form of treating cancer cells in brain diseases (Akimoto, 2016). Three years later, Akimoto revisited PDT in combination with talaporfin in a clinical trial. The research group consisted of 47 patients. The results of the experiments also confirmed that talaporfin is an effective and useful photosensitizer (Akimoto et al., 2019). Miki et al., in turn, developed the NPe6-PDT nanocomplex in order to assess the effectiveness of the therapy. *In vitro* studies were performed on glioblastoma cell lines. Analysis of mitochondrial cell death pathways and cellular responses showed that the cell death observed was a result of the therapy. Cell morphology showed necrosis, which was a function of exposure time and dose (Miki et al., 2013). Figure 6 shows the structure of Talaporfin

#### 3.1.6 Hypericin, HYP

One of the strongest natural photosensitizers used in photodynamic therapy is hypericin (HYP). It is commonly found in plants called St. John’s wort. Hypericin is a potential clinical anti-cancer agent as many studies have shown its strong anti-cancer effects. Its effectiveness depends on the type, concentration and dose of light applied (Agostinis, et al., 2002). Many studies have confirmed that HYP selectively accumulates in the neoplastic tissue of tumors. From scientific reports it can be concluded that chemotherapeutics significantly improves the already relatively high effectiveness of hypericin-supported PDT (Dong et al., 2021). Hypericin is the natural active ingredient of *Hypericum perforatum* (St. John’s wort). Hypericin has additional pharmaceutical and medical applications as it exhibits antidepressant, anticancer and antiviral effects (Liu et al., 2020). *Hypericum perforatum* is also a popular herbal remedy recommended for the treatment of depression. Due to the progressive rate of utilization of St. John’s wort on a global scale, it is one of the conventional antidepressants (Ng et al., 2017). Thanks to its active ingredients, hypericin is used in the treatment of mental and neurodegenerative disorders (Zirak, et al., 2019). Hypericin contains anthraquinone derivatives (hypericin and pseudohypericin), flavonoids, prenylated floroglucinols (hyperforin), tannins, phenolic acids and volatile oils. It has calming and astringent properties. It is used for excitability, neuralgia, anxiety and as a nerve tonic. St. John’s wort has a long history of traditional use in topical preparations for wound healing. It is now used in homeopathic products as well as in herbal products (Zirak, et al., 2019).

Hypericin, a natural small molecule extracted from *H. perforatum*, has been studied as a photosensitizer for photodynamic therapy and as an effective agent for necrosis targeted imaging and tumor therapy. Although the molecular targeting sites of hypericin are still under investigation, applications of hypericin for necrosis contrast agent development and necrosis targeted radiotherapy have increasingly gained attention. The development of an effective and efficient hypericin application model with a high safety standard is a challenge for the coming years (Rizzo et al., 2019; Han et al., 2020). In cases of brain tumors, hypericin has been used, among others, by Misuth et al., (Misuth et al., 2017). Rottlerin was also used in this study to test their synergistic effect. Cells from the U-87 MG line were treated in different ways: only hypericin, only rottlerin, or a combination thereof. The results showed that cells after exposure to laser light and after application of hypericin or its combination with rottlerin significantly reduced the metabolic activity of the cells’ mitochondria. In turn, irradiation of cells treated only with rottlerin did not reduce their mitochondrial metabolic activity. Additionally, after PDT, cells treated with 0.5 µM hypericin, 2 µM rottlerin and their combination significantly reduced the population of living cells.

**FIGURE 7:**
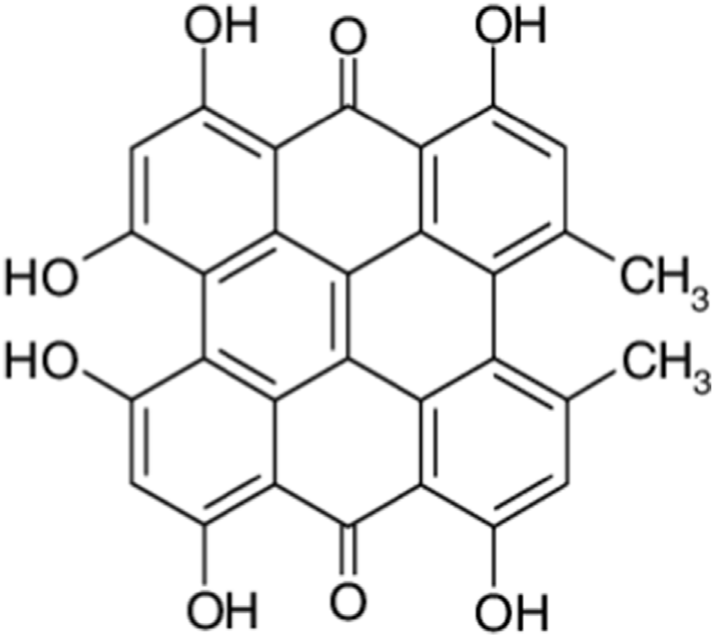
Structure of hypericin.

Figure 7 shows the structure of Hypericin.

#### 3.1.7 Phthalocyanines

Phthalocyanines are another group of photosensitizers used in PDT. These are aromatic, heterocyclic compounds. Its structure has 4 aromatic rings. They absorb light between 650 and 850 nm. Research has been conducted for several years on the use of phthalocyanine photosensitizers in PDT. Currently, they are being tested to increase selectivity, improve the process of accumulation in cancer cells, and in the development of new synergistic therapeutic strategies to increase the effectiveness of PDT.

Phthalocyanines undergo many types of chemical transformations, such as: coordination, polymerization, aggregation, oxidation and reduction reactions; they take an active part in the processes of catalysis, and photochemical transformations (Li et al., 2023).

Phthalocyanines have relatively low toxicity in the dark. However, after exposure to laser light, they become extremely photobiologically active (de Siqueira et al., 2022). Phthalocyanines, as a group of second-generation photosensitizers, have enhanced photochemical and photophysical properties compared to first-generation photosensitizers. The most important features characterizing phthalocyanines are their unique spectroscopic, luminescent (fluorescence and phosphorescence), magnetic (para- and diamagnetism) properties, as well as their thermal stability, photoconductivity, photoemission and surface activity. Phthalocyanines are very reactive compounds that undergo various types of reactions - coordination, polymerization, aggregation, formation of acids, bases and salts, redox reactions, catalysis, sorption and photochemical reactions (Santos et al., 2020). It turns out, for example, that phthalocyanine photosensitizers have a greater tendency to create radical species, while porphyrin photosensitizers tend to generate singlet oxygen.

Phthalocyanines have lipophilic elements in their structure, which makes their application in clinical trials difficult. To overcome this limitation, scientists are designing systems and platforms that facilitate their delivery (Miretti et al., 2021).

Conjugation of phthalocyanines with diamagnetic elements such as zinc, aluminum and silicon seem to improve the efficiency of singlet oxygen generation. Phthalocyanines combined with zinc are characterized by low toxicity in dark conditions. They are also characterized by a high therapeutic effect and low sensitivity of the skin and other organs. The disadvantage of this type of photosensitizer is poor water solubility. In turn, third-generation zinc (II) phthalocyanines, i.e., the so-called a second-generation photosensitizer, have been designed in combination with a carrier. Currently, photosensitizers with a carrier based on liposome, polymer micelles and nanoparticles are proposed in the literature. The use of this type of carrier improves the bioavailability of the applied photosensitizers and their transport to target sites. Roguin et al. attempted to analyze various liposomes and polymer micelles as carriers for photosensitizers based on zinc (II) phthalocyanines. Their research confirmed that in combination with carriers based on both liposome and polymer micelles, photosensitizers were more soluble. In the case of photodynamic activity, micelles were characterized by a higher activity coefficient towards cancer cells (Roguin et al., 2019).

**FIGURE 8:**
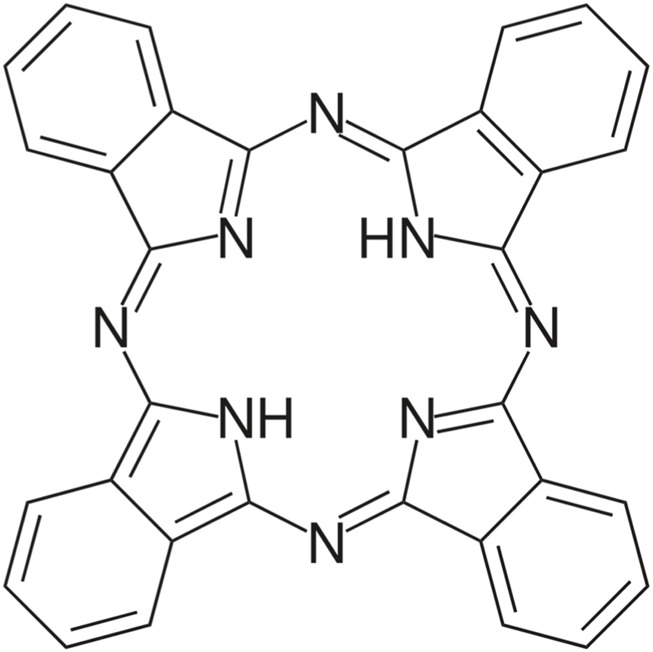
Structure of phthalocyanines.

In the treatment of brain tumors, phthalocyanines have been used, among others: by Castilho-Fernandes et al. (Castilho-Fernandes et al., 2017). The authors conducted an *in vitro* study using the U-87 MG line. The experiment used chloroaluminum phthalocyanine encapsulated in a new drug delivery system and designed as a nanoemulsion. After applying the nanomulsion to the cells and after exposure to laser light, the changes were observed under a microscope. The results were as follows: cells irradiated with 70 mJ/cm^2^ energy showed 56% ± 13% cell death, while doses of 140 and 250 mJ/cm^2^ caused 34% ± 15% and 33% ± 4% cell death, respectively, compared to untreated cells and control cells. So the dose of laser light was not directly proportional to the number of lethal cells. After PDT, glioma cells were clearly smaller and shrunken compared to cells from the control group. The results indicate that the proposed treatment model is effective and induces apoptosis. Figure 8 shows the structure of Phthalocyanines.

In turn Table 2 summarizes information regarding individual photosensitizers.

**TABLE 2:** Characteristics of photosensitizers used in the treatment of brain tumors with PDT.

Types of photosensitizer	The wavelength of use for each photosensitizer [nm]	Model/Tumor cell lines	Optimal use concentration	Light dose	References, years
Porfimer sodium, Photofrin, HPD	632	U-87 MG, U25ln, glioma cells	application of different doses (low doses in the amount of: 2.5 μg/mL and 1 μg/mL)	100 mJ/cm^2^	Jiang et al. (2002)
	630	U-87 MG cells	12.5 mg/kg	17 J/cm^2^	Jiang et al. (1998)
	635	Mouse Model, U-87 MG cells	2 mg/kg	80 J/cm^2^	deCarvalho et al. (2007)
	630	U-87 MG cells	1 μg/mL	cells were exposed to different light doses (0 J/cm^2^, 0.8 J/cm^2^, 1.3 J/cm^2^	Lee et al. (2010)
	630	canine glioma model	application of different doses (ranging from 0.75 to 4 mg/kg)	1800 J	Whelan et al. (1993)
5-aminolevulinic acid, 5-ALA	635	Interstitial PDT	20 mg/kg	median energy dose: 8996 J	Lietke et al. (2021)
	635	DAOY, UW228, PFSK-1, BT16 cell lines	varying concentrations: (25, 50, 75, and 100 μg/mL)	25 J/cm^2^	Schwake et al. (2018)
	635 nm	HGG13, HGG30, HGG146, HGG157 and HGG1123	0.3 mM	0–27 J/cm^2^	Omura et al. (2023)
Temoporfin (Foscan), m-THPC	652	C6 glioma cell lines	0.5 μgm/L	29 J/cm^2^ and 58 J/cm^2^	Bœuf-Muraille et al. (2019)
	558	C6 glioma cells	different dose: 0.3 mg/kg, 0.02 mg/kg, 0.015 mg/kg	-	Mannino et al. (2008)
	652	Intraoperative PDT	0.15 mg/kg	20 J/cm^2^	Vanaclocha et al. (2015)
Chlorin e6	665	GL261 cells	different dose: 0, 0.625, 1.25, 2.5, 5, and 10 μg/mL	5 J/cm^2^	Teng et al. (2020)
Talaporfin (LS11, MACE, NPe6)	664	Intraoperative PDT	40 mg/m^2^	27 J/cm^2^	Muragaki et al. (2013)
	664	Rat glioma model	5.0 mg/kg	10 J/cm^2^	Namatame et al. (2008)
	664	Intraoperative PDT	40 mg/m^2^	27 J/cm^2^	Akimoto et al. (2012), Akimoto et al. (2019)
	664	T98G cells	0–50 μg/mL	10 J/cm^2^	Miki et al. (2013)
Hypericin, HYP	590	U-87 MG	0.5 μM	4 J/cm^2^	Misuth et al. (2017)
Phthalocyanines	650	U-87 MG	1 μmol	70, 140, and 250 mJ/cm^2^	Castilho-Fernandes et al. (2017)

### 3.2 Clinical and preclinical studies of PDT in brain tumors–a review

Currently, PDT is undergoing intensive clinical trials (Kostron, 2010). The beginnings of PDT treatment of patients with brain cancer dates back to the 1980s. One of the first cases in which PDT was administered was in treatment of glioblastoma multiforme. During the procedure, resection of the glioblastoma cavity was performed. The photosensitizers used were porfimer sodium, 5-ALA and temoporfin. The light sources used were: dye lasers, diode lasers and lamps. The results of the therapy confirmed the hypotheses that PDT may be helpful in the treatment of brain tumors. Currently, PDT is being used in combination with other compatible therapies. The methods of adjuvant treatment include intraoperative diagnostics by means of photodetection (PD) and fluorescence-guided resection (FGR) (Stylli et al., 2005). An example of the application of PDT in the treatment of glioblastoma was the research conducted by Muller and Wilson. The authors presented the results of research conducted in Toronto, Canada. The study group consisted of 96 patients with supratentorial glioma who underwent PDT treatment with porfimer sodium (Muller and Wilson, 2006). During the research, the authors were aware that there was little clinical or even preclinical data related to the treatment of brain tumors with PDT. The aspect of tumor infiltration into adjacent tissues is extremely important. Achievements in the field of focused radiation are crucial in implementing further clinical trials (as the authors emphasize). This aspect is extremely important in the context of further clinical trials.

In turn, Stummer et al. (Stummer et al., 2006) presented the results of research conducted in Germany. The research group consisted of 322 patients diagnosed with malignant glioma. The photosensitizer used was 5-ALA. The aim of the study was to evaluate the effect of surgical resection with 5-ALA application. The results showed that during resection with the application of 5-ALA, the tumor was completely removed in 65% of patients. However, without the application of 5-ALA (only white light) the tumor was completely removed in only 36% of patients. It is worth noting that the authors were aware that maximum cytoreductive treatment of malignant gliomas is beneficial for patients, but the issue is still the subject of numerous discussions. The authors report that neurosurgeons are increasingly attempting to remove tumors under contrast enhancement. However, it is not 100% effective and such a procedure is only achieved with a small number of patients. To enhance this effect, intraoperative MRI, neuronavigation or ultrasound are often used.

An aim of the research conducted by Eljamel et al. was characterization of PDT in the treatment of glioblastoma in comparison with fluorescence-guided resection. The article clearly shows that both applied methods significantly prolonged patient’s lives (Eljamel et al., 2008). The main challenge of this study was the fluorescence of the photosensitizer Photofrin, because it is very similar in color to the background. The combination application of photosensitizers, i.e., ALA-induced PpIX and Photofrin^®^, eliminated this limitation. Potential side effects of the test include a temporary increase in liver enzyme levels. In the study by (Eljamel et al., 2008), these enzymes were not monitored and no clinically significant abnormalities in liver or kidney function were observed. Nevertheless, as the authors encourage, the presentation of the study is an encouragement to implement further test research protocols that have improved the therapeutic procedure.

In a study by Kostron and colleagues, 26 patients diagnosed with recurrent glioblastoma multiforme were enrolled in a PDT study with Foscan^®^. Before treatment, all tumors progressed and standard therapeutic options (irradiation, chemotherapy) were exhausted. After aggressive fluorescence-guided resection, intraoperative PDT was performed. Compared to matched controls, significantly better survival was demonstrated in the treatment group (Kostron et al., 2006; Kaneko et al., 2018). According to the authors, several specific side effects were observed in patients, such as increased skin sensitivity and swelling. In the context of further research, certain schemes can be developed that do not eliminate the presented side effects.

In a study by Han-Wen Guo et al., PDT was applied to a mouse model of human glioblastoma using an organic light-emitting diode and a single dose of 5-aminolevulinic acid (ALA) as a photosensitizer. Tumor volume was measured by bioluminescence imaging and the survival time of the animals was recorded. It was shown that in animals with a similar tumor volume before and immediately after irradiation, the average overall survival time of mice after PDT was significantly longer than in control mice (Guo et al., 2015). Testing metronomic PDT in small animals raises several problematic issues related to the maintenance and transportation of the lighting device. The authors used organic LEDs for this purpose. According to the authors, the study confirmed the feasibility of low fluence rate and long duration of ALA-PDT treatment using OLED without animal anesthesia. However, also taking into account such characteristics as dosimetric indicators, including photosensitizer fluorescence and the degree of tissue oxygenation, would enable understanding of certain therapeutic limitations and improve future treatment regimens.

In a 2013 study by Yoshihiro Muragaki et al., the potential efficacy and safety of iPDT with sodium talaporfin and semiconductor laser irradiation in patients with primary malignant parenchymal brain tumors was investigated. A single intravenous injection of talaporfin sodium was administered to 27 patients with suspected newly diagnosed or recurrent primary malignant parenchymal brain tumors 1 day before resection. Twenty-two patients with a histopathologically confirmed diagnosis of primary malignant parenchymal brain tumor were enrolled in the study. Intraoperative PDT with the use of sodium talaporfin and a semiconductor laser may be considered a potentially effective and sufficiently safe option for the adjuvant treatment of primary malignant parenchymal brain tumors (Muragaki et al., 2013).

One of the tests of newly developing therapies are preclinical studies. The conducted studies enable the selection of appropriate therapeutic regimens including light dose. According to the literature, longer exposure of laser light to lesions may increase the effectiveness of PDT in the brain in combination with other supportive therapies, such as LED light delivery (Kostron, 2010).

Preclinical studies also include *in vitro* studies on cell lines. An example is research on glioblastoma cell lines that were treated with PDT using HYP. Studies have confirmed that PDT with HYP was highly effective in reducing the number of glioblastoma cells and had an inhibitory effect on cell growth and reproduction. The cited studies indicate that hypericin-assisted PDT may become an effective method of treatment in various oncological diseases (Dong et al., 2021).

Despite the many advantages of PDT and its high efficiency in the treatment of brain tumors, there are still doubts about its use as a standard adjuvant therapy. The effects of PDT in both clinical and preclinical trials are satisfactory, however, additional studies targeting brain tumors are still needed to help take into account the pharmacokinetic aspects (Kim and Lee, 2022). Although there have been numerous studies demonstrating the use of PDT in the treatment of brain tumors (including malignant tumors), most of them are in the early stages. In order to effectively assess PDT, it is necessary to introduce certain criteria, such as: tumor subtype, supportive techniques, light sources or type of photosensitizer. Currently, it is difficult to obtain a general understanding of the current state of knowledge and assess the direction in which the treatment of malignant brain tumors with PTD is heading (Quirk et al., 2015).

The clinical studies described previously were described in publications of reputable journals. However, based on the publicly available clinical trials database ClinicalTrials.gov, nine clinical trials were registered in which patients with brain tumors were subjected to photodynamic therapy. The clinical studies cited are summarized in table 3 presenting their detailed characteristics. The number of registered clinical trials on the treatment of brain tumors with PDT is not large. This demonstrates the complexity of such tests, potential complications and long time durations. Table 3 presents the characteristics of 9 clinical trials officially registered in the ClinicalTrials.gov database. The search words were: condition: brain tumor, intervention: photodynamic therapy.

**TABLE 3:** Registered clinical trials in the database ClinicalTrials.gov.

Countries	Study type	Phase	Official title	Conditions	Interventions	Years and status
Milwaukee, Wisconsin, United States	Interventional	2	A Phase II Study of Photodynamic Therapy (PDT) With Photofrin^®^ (IND 104,613) For Recurrent High Grade Gliomas in Adults	The photosensitizer applied was Photofrin^®^ (porfimer sodium) (Pinnacle Biologics, Inc., Bannockburn, IL, United States) at a dose of 2.5 mg/kg 24 h before planned surgical resection	Standard brain tumor surgery + PDT	2015–2017 Terminated with Results
				The tumor was exposed to red laser light. The total light dose was 240 J/cm^2^		
				The light was delivered to the brain via a fiber optic cable. In order to distribute the light evenly, a knob is placed at the end of the optical fiber		
Wauwatosa, Wisconsin, United States	Interventional	1	Photodynamic Therapy (PDT) for Poor Prognosis Recurrent/Refractory Malignant Brain Tumors - A Phase I Study	The photosensitizer used was Photofrin (porfimer sodium). The dose was gradually increased in the study. The photosensitizer was administered 24 h before resection and PDT. The light was delivered to the brain via optical fiber. The purpose of the Intralipid applied was to disperse light and provide even coverage	Standard brain tumor surgery + PDT	2013–2018 Completed
Buffalo, New York, United States	Interventional	1	Phase I Study of the Safety of Intracavitary Photodynamic Therapy (PDT) of the Brain Bordering Resected Recurrent Glioblastoma or Gliosarcoma Using Intravenous Photobac^®^ and a Balloon Light Applicator	The photosensitizer used was Photobac^®^, which was administered 24 h before the procedure. During treatment, the dose will be increased in 8 steps	Standard brain tumor surgery + PDT	2023–2026 Recruiting
				The light source used was a laser with an energy of 50 J/cm^2^ and a wavelength of 787 nm		
Englewood, Colorado, United States	Interventional	3	Prospective Clinical Trials in the Use of Photodynamic Therapy (PDT) for the Treatment of Malignant Supratentorial Brain Tumors	The intravenously administered photosensitizer was Photofrin (porfimer sodium). No information about the wavelength of laser light or the type of laser. Exposure time: 5 days a week for 5–6 weeks. The group of patients was also divided depending on the dose. Additionally, patients with a lower light dose received chemotherapy with procarbazine	Standard brain tumor surgery + PDT + chemotherapy	1998- no data Unknown status
Buffalo, New York, United States						
Pittsburgh, Pennsylvania, United states						
Toronto, Ontario, Canada						
Cleveland, Ohio, United States	Interventional	3	A Randomized Prospective Two Arm Clinical Trial of High Light Dose and Low Light Dose PDT in the Treatment of Recurrent Malignant Supratentorial Gliomas Using Porfimer Sodium [Photofrin]	The photosensitizer used was Photofrin (porfimer sodium)	Standard brain tumor surgery + PDT	2005–2006 Completed
				No information about the wavelength of laser light or the type of laser. The patients were divided into two groups. The differentiating factor was the dose of laser light		
Milwaukee, Wisconsin, United States	Interventional	1	Photodynamic Therapy for Childhood Brain Tumors, A Phase I Study	The photosensitizer used was Verteporfin. The study aimed to determine the maximum tolerated dose of a photosensitizer. No information about the wavelength of laser light or the type of laser	Standard brain tumor surgery + PDT	1994- no data Unknown status
Seattle, Washington, United States	Interventional	1	A Phase 1 Study of Aminolevulinic Acid (ALA) to Enhance Visualization and Resection of Malignant Glial Tumors of the Brain	The photosensitizer used was aminolevulinic acid. The aim of the study was to determine the optimal dose of photosensitizer	Standard brain tumor surgery with intra-operative frameless MRI stereotactic guidance and intra-operative ultrasound guidance	2010–2012 Terminated
				The photosensitizer was administered orally 4 h before surgery		
				No information about the wavelength of laser light or the type of laser. Patients were monitored with MRI within 48 h of completion of surgery		
Taipei, Taiwan	Interventional	2	Prospective, Open-labeled, Clinical Trial in Compassionate Use to Evaluate the Efficacy and Safety of Photodynamic Therapy in the Treatment of Malignant Intracranial Tumors	The photosensitizer administered intravenously was Photosan^®^ at a dose of 2 mg/kg. Red halogen light with a wavelength in the range of 625–635 nm was used. Irradiation dose with an energy density of 100 J/cm^2^ with a power density of 500 mW/cm^2^ to 600 mW/cm^2^		2009- the study was withdrawn
Guayaquil, Guayas, Ecuador	Observational	-	5-Aminolevulinic Acid (5-ALA) Gliolan^®^: Usage Increase Proposal for Neurosurgical Procedures in High-Grade Gliomas	Patients will be administered Gliolan^®^ orally 3 hours (range 2–4 h) before anesthesia. No information about the wavelength of laser light or the type of laser	Fluorescence-Guided Surgery using 5-aminolevulinic acid (5-ALA)	2023–2026 Not yet recruiting

## 4 Therapeutic challenges in PDT

### 4.1 Light sources

The main challenge in PDT is how to apply the light dose so that it is applied evenly and at the right depth to the treated area. In addition, the form of application of the photosensitizer and the method and time of accumulation in tissues are a challenge. Therefore, light and photosensitizer dosages are extremely complex and are key factors that determine the success of treatment (Quirk et al., 2015). The first sources of laser light were argon and xenon lasers (Quirk et al., 2015). Currently, laser and non-laser sources are applied. The group of laser light sources includes argon lasers, dye lasers, solid-state lasers, optical parametric oscillators and diode lasers. The group of non-laser light sources includes halogen, xenon, metal halide, sodium, fluorescent lamps as well as light-emitting diodes and femtosecond solid-state lasers (Gunaydin, et al., 2021). Choosing the right light source and the way it is applied is one of the challenges in PDT. Depending on the applied photosensitizer, red or blue light i useds, which have different penetration depths (Gunaydin, Gedik, Ayan, 2021). Currently, in therapy, light sources with a longer wavelength and greater penetration are sought, which can be used to treat internal organs. Light with absorption wavelengths between 600 and 800 nm are most often used (Cramer and Chen, 2020; Gunaydin, Gedik, Ayan, 2021). Photosensitizers also require the presence of appropriate oxygen species to be active (Gunaydin et al., 2021).

### 4.2 Hypoxic environment

One of the challenges of modern medicine is to increase the effectiveness of cancer treatment, which is one of the main causes of morbidity and mortality in people around the world. Despite the enormous amount of work of scientists, overcoming cancer still seems distant. The obstacle is not only the lack of effective compounds, but even those that show promising anticancer properties encounter additional difficulties that are not always taken into account in model studies and quite rarely in preclinical studies. One such aspect that may be responsible for the reduced effectiveness of PDT is the lack of oxygen in the tumor tissue, called hypoxia, which characterizes the microenvironment of solid and hematological tumors. Hypoxia has a double negative role. On the one hand, it may lead to the formation of a more aggressive phenotype of cancer cells, which are invasive and contribute to the formation of metastases. On the other hand, it reduces the effectiveness of oxygen-dependent chemotherapy and radiotherapy, as well as hinders the delivery of drugs to the tumor due to abnormal microcirculation. All these aspects encourage us to take a closer look at the phenomenon of hypoxia in cancer. To do this, you need, among others, markers that will identify areas of hypoxia. On the other hand, it is important that the effectiveness of the tested compounds as potential anticancer drugs is also checked under hypoxic conditions.

At the moment, there are few literature reports on how to deal with hypoxia in photodynamic therapy for the treatment of glioblastoma. The reason for this phenomenon may be the aspect of the complexity of this process, and its role and impact on PDT. Most of the publications describing hypoxia and its impact on the effectiveness of PDT are articles from 2021-2022, which confirms that this topic has been analyzed relatively recently. Additionally, research is still ongoing presenting the characteristics of this phenomenon in the context of PDT treatment and proposing solutions to limit its impact on the effectiveness of PDT.

In the field of hypoxia, research was also conducted by Ihata et al. In this experiment, they managed to confirm that the effectiveness of 5-ALA PDT under hypoxic conditions was not significantly reduced, even though the content of protoporphyrin produced was lower compared to standard PDT treatment conditions. The authors claim that oxygen concentrations below 5% initiate a series of cellular responses that induce hypoxia. According to the authors, the inclusion of various types of inhibitors in PDT will enhance the therapeutic effect of the destruction of brain tumor cells. Further studies (mainly *in vitro*) illustrating different variants of the tumor environment (with reduced or increased oxygen content) are necessary in order to select the most appropriate therapeutic method. Individual *in vitro* results do not make it possible to apply the presented innovative methods to clinical trials. Therefore, further research using cell lines and animal models testing ALA-PDT is needed (Ihata et al., 2022).

Albert et al., in an *in vitro* study, used two glioblastoma cell lines to analyze and characterize the effect of the level of oxygen partial pressure on the physiology of cancer cells and the effectiveness of PDT. The experiment showed that changing the partial pressure of oxygen from 19% to 9% did not change the intracellular accumulation of PpIX. However, reducing partial pressure changed the reactivity of glioma cells of 1 cell line. The therapeutic efficacy in the treatment of U-87 MG cells was not satisfactory at a pressure of 9%. The treatment of these cells required a pressure of 20% and a longer exposure time. This experiment illustrates that precise control of oxygen concentration is necessary, both *in vivo* and *in vitro* (Albert, Hefti, Luginbuehl, 2014).

Sunil et al., in order to reduce the impact of hypoxia proposed enhanced combination therapy using light-responsive oxygen generators that capture antigens. The designed model contained the Nutlin-3a molecule and the photosensitizer PpIX. Such a model can release oxygen when activated with laser light of a specific wavelength. The project assumes that generating oxygen on demand in hypoxic conditions leads to an increase in the tumor oxygenation level. By eliminating the phenomenon of hypoxia and stabilizing the level of partial pressure and oxygen concentration, the effectiveness of PDT may be higher (Sunil et al., 2021).

The ideas presented above to improve tumor oxygenation and thus improve the effectiveness of PDT are pioneering research. There is still a need to conduct research and thus implement further ideas that will be used in vivo research.

## 5 Conclusion

The science of photodynamic therapy has been on going for over 100 years, and its application in clinical practice began at least 35 years ago. Over the past 20 years, knowledge about the operation and use of PDT has greatly expanded. New proposals for photosensitizers, light sources and light delivery methods are some of the possibilities to improve this treatment technique. Molecular strategies based on nanotechnology are constantly being developed to increase the effectiveness and selectivity of PDT. Photodynamic therapy is a highly selective method of treating various malignant tumors of the skin and internal organs. It allows patients to avoid many adverse side effects associated with the use of such forms of treatment as radiotherapy or chemotherapy. Photodynamic therapy is characterized by a favorable profile of side effects, and the ability to enhance the anti-cancer immune response. At the moment, PDT has not yet reached its maximum potential due to the limitations of the classic photosensitizers used, including, i.e., light absorbance, penetration depth and cellular uptake. It is expected that intensive research on the use of photodynamic therapy in the treatment of cancer, including brain cancer, will soon make PDT one of the world standards of therapy in the treatment of these diseases.
